# The do-not-resuscitate order: incidence of documentation in the medical records of cancer patients referred for palliative radiotherapy

**DOI:** 10.3390/curroncol13020003

**Published:** 2006-04

**Authors:** N.M.E. Bradley, E. Sinclair, C. Danjoux, E.A. Barnes, M.N. Tsao, M. Farhadian, A. Yee, E. Chow

**Affiliations:** * Rapid Response Radiotherapy Program and Bone Metastases Clinic, Toronto–Sunnybrook Regional Cancer Centre, University of Toronto, Toronto, Ontario

**Keywords:** Advance directives, cardiopulmonary resuscitation (cpr), do-not-resuscitate (dnr), end-of-life ethics, prehospital dnr orders

## Abstract

Patients with symptomatic metastases referred for outpatient palliative radiotherapy for symptom control at the Rapid Response Radiotherapy Program (rrrp) and the Bone Metastases Clinic (bmc) at the Toronto–Sunnybrook Regional Cancer Centre have a limited life expectancy. Relevant medical information is missing from the files of many referred patients when they arrive at the clinics, potentially causing delayed treatment and ambiguity in the best management of their needs in situations of worsening condition. Clear documentation of the do-not-resuscitate (dnr) order is imperative to avoid panic and the taking of unnecessarily aggressive measures in situations in which cardiopulmonary resuscitation (cpr) has no benefit or is not desired. Here, we report the current practices of cpr code status documentation for patients referred to the rrrp and the bmc for out-patient palliative radiotherapy.

We reviewed referral notes and accompanying medical records for 209 consecutive patients seen in the rrrp and the bmc during May–August 2004 for documentation of cpr-related advance directives. Patient demographics and cancer history were also recorded.

Only 13 (6.2%) of the 209 patients had any documented reference to cpr code status. Of these 13 patients, 8 were dnr-coded, and 5 were full code. As compared with patients having no documented cpr code status, patients with documented status were significantly older (median age: 77 years; *p* = 0.0347), had poorer performance status (median Karnofsky performance status score: 40; *p* = 0.0001), and were more likely to be referred hospital inpatients (69%, *p* = 0.0004).

Only a small proportion of symptomatic advanced cancer patients had any documentation of cpr code status upon referral for outpatient palliative radiotherapy. In future, our clinics plan to request information about cpr code status on our referral form.

## 1. INTRODUCTION

Resuscitation of patients with sudden cardiopulmonary arrest was first implemented in the 1960s^1^. Cardiopulmonary resuscitation (cpr) may be desirable in trying to prevent sudden, unexpected death in patients whose medical condition would be expected to improve following successful cpr. However, cpr is rarely successful, especially in patients with advanced metastatic cancer. Patients who undergo cardiopulmonary arrest have a 3%–14% chance of survival if the arrest occurs outside the hospital and a 10%–20% chance if the arrest occurs in hospital^2^.

Since the 1970s, documentation of the do-not-resuscitate (dnr) order has been in practice in situations in which cpr is thought to have no valuable role and little chance of success—especially in patients with an underlying incurable medical condition such as metastatic cancer^3^–^7^. Potentially life-prolonging treatment is withheld in patients when the chance of recovery or improvement from the underlying illness is thought to be very low. However, despite the use of the dnr order in the thirty or so years since the emergence of cpr, many difficulties about how dnr decisions are put into practice remain, including the use of consistent and clear documentation in medical records.

Patient care policy at Sunnybrook and Women’s College Health Sciences Centre (swchsc) states that, unless a not-for-resuscitation or dnr order is documented in the patient’s chart and medical records, cpr must be initiated on every patient in the hospital who undergoes cardiopulmonary arrest, even if the death is expected.

**Figure 1:**
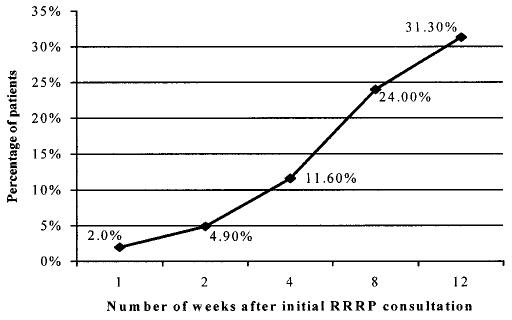
Cumulative mortality after initial consultation at the Rapid Response Radiotherapy Program clinic (rrrp), 1999–2001.

The Toronto–Sunnybrook Regional Cancer Centre (tsrcc) of swchsc is one of two regional cancer centres offering radiotherapy in the greater Toronto area. The Rapid Response Radiotherapy Program (rrrp) and the Bone Metastases Clinic (bmc) at the tsrcc offer quick access to palliative radiotherapy on an outpatient basis to relieve symptoms and improve quality of life for people with symptomatic metastatic cancer^8^. Cancer patients seen in the rrrp and bmc commonly have multiple and varying symptoms, poor performance status, multiple comorbidities, and a limited life expectancy; they are therefore at risk of cardiopulmonary arrest. The median survival of the 830 patients seen in the rrrp between 1999 and 2002 was 4.1 months (122 days)^9^. Of these 830 patients, 17 (2.0%) died 1 week after their initial consultation, and a cumulative 41 (4.9%) died at 2 weeks, 96 (11.6%) at 4 weeks, 199 (24.0%) at 8 weeks, and 260 (31.3%) at 12 weeks (3 months) after their initial consultation^9^ (Figure 1).

The mandate of the two clinics is to shorten delays and waiting time in the provision of palliative radiotherapy to within 1 week of referral for patients with a prognosis of less than 12 months. When appropriate, consultation, simulation, radiotherapy planning, and treatment delivery occur on the day of the first visit so as to minimize multiple burdensome trips to the clinic for the patient. If treated on the same day as the initial consultation, patients are typically in the cancer centre for several hours (the time required for treatment planning, dosimetry calculations, and treatment delivery^8^). Because patients are in the cancer centre for most of the day, the risk of cardiopulmonary arrest in the centre is great.

Because the tsrcc is an outpatient oncology clinic, most patients are referred from physicians in the community with appropriate medical documentation and reports. Approximately one quarter of patients seen in the rrrp are hospital inpatients at the time of their rrrp and bmc consultation 8. Inpatients of health care institutions are generally more symptomatic and have poorer performance status scores than do patients coming from home^10^. We therefore ask that the inpatients of other health care institutions be accompanied by a nurse escort, and we require that they come with their inpatient hospital chart and all relevant medical documentation. However, missing patient medical information at the time of initial consultation—including imaging, imaging reports, medication lists, and inpatient hospital charts—is a common presenting clinical scenario in the rrrp.

Full medical documentation is essential for treatment planning and for providing the best possible care for patients while they in the clinic. Missing or unclear medical documentation not only potentially delays the simulation of, and treatment with, palliative radiotherapy, it can also can be potentially hazardous to the patient.

In another common clinical scenario, inpatients frequently arrive at the rrrp without a nurse, further adding to the risk of confusion in situations of missing information.

In an outpatient radiotherapy clinic where newcomers are usually unknown to the health care providers, proper documentation of advance directives is essential in the event of cardiopulmonary arrest. If a patient has chosen not to be resuscitated, but no documentation is provided, that patient may be unnecessarily put through aggressive measures that he or she chose in advance to refrain from.

Here, we report the current practices of dnr documentation in cancer patients with metastatic disease referred to the outpatient palliative radiotherapy clinics at the tsrcc. We also explore differences in the documentation of life-sustaining treatment decisions between the inpatient and outpatient subpopulations.

## 2. PATIENTS AND METHODS

We reviewed the referral information and medical forms for all consecutive patients seen in the rrrp and bmc clinics at tsrcc during May–August 2004 for documentation of cpr-related advance directives, if any. All medical documents, including progress notes, imaging reports, cancer history, previous treatment records, and ambulance transfer forms, were reviewed. Inpatient charts from other health care institutions, if available, were also reviewed for all in-patients attending the clinics. Patient information was collected at the time of the first visit during the 4-month period. Patients were included in the study only once—that is, follow-up visits, if any, were not recorded.

We collected data on patient demographics, including age, sex, Karnofsky performance status (kps) score^11^ at time of consultation, and disease information. Data about the disease included the primary cancer site and its extent. We collected data on whether the patient came from home, hospital, hospice, or nursing home, and whether the patient came to the clinic by ambulance. We also collected information regarding advance directives from the chart documentation review, including whether documentation of cpr code status was present in the medical records, whether the patient was a cpr full code or dnr code, the type of physician that documented the cpr code status, the location of the advance directive in the records, and whether the patient and family had been involved in the decision. Patients who were identified as “cpr full code” were patients who would undergo cpr during cardiopulmonary arrest; patients classified as “dnr code” were patients who had decided not to have cpr and would not undergo an attempt at resuscitation.

Ethical approval for the study was obtained from the hospital research ethics board.

### 2.1 Statistics

Descriptive statistics were recorded as percentages for proportions and as means, medians, standard deviations, and ranges for continuous variables. For unordered categorical variables (sex, number of metastasis sites, and the presence of bone, brain, liver, lung, lymph-node, or soft-tissue metastases), we used the chi-square test to detect significant differences in the proportions of patients with cpr documentation. We used the Fisher exact test to detect non-random associations between categorical variables that did not meet the minimum sample size of 5 in each group for the chi-square test. We also used the Fisher exact test to detect differences in the cpr documentation between the inpatient and outpatient subpopulations. All continuous variables were tested using the Student *t-*test, including age and kps score. Tests were considered significant if the *p* value was less than or equal to 0.05, and all tests were two-sided. All data were analyzed using the Statistical Analysis System (SAS: SAS Institute, Cary, NC, U.S.A.).

## 3. RESULTS

During May–August 2004, a total of 209 patients were seen in the rrrp and the bmc. The proportions of men and women seen in the clinic were approximately equal. The median age of the patients was 70 years (range: 28–96 years). The median kps score was 70 (range: 20–100). Most of the patients came from home (76.6%), and approximately one quarter arrived at the clinics by ambulance. The most common primary cancer sites were lung, breast, and prostate. More than half of the patients (57.9%) had multiple sites of metastasis, and most patients had bone metastases (76.6%). Table I summarizes the patient demographics and disease information.

Based on the medical records, physician progress notes, inpatient hospital charts, and ambulance transfer forms that were available at the time of clinic visits, only 13 (6.2%) of the 209 patients had any documented reference to cpr code status. Of these 13 patients, 8 were dnr coded. The other 5 patients were full code and would request full ventilation and aggressive measures in a medical emergency situation.

Most cpr documentation was found in the physician progress notes and was documented by the patient’s medical oncologist. Details about the cpr discussion and decision were ambiguous. Records stated that 3 patients made the decision themselves and that, for 2 patients, a family member acted as substitute decision-maker because of patient incompetence. Who made the cpr code status decision for the other 8 patients was unknown. Table II summarizes the details of the cpr documentation for the 13 patients.

A total of 8 patients (61.5%) with cpr documentation arrived at the clinic by ambulance. Of the 13 patients with cpr documentation, 9 patients presented at the clinics with an oncologic emergency: 7 with spinal cord compression, 1 with impending spinal cord compression, and 1 with superior vena cava obstruction. Another patient of the 13 presented with a pathologic fracture. Of the 13 patients with documented cpr code status, 3 patients had lung cancer, 2 had breast cancer, 2 had prostate cancer, 1 had an unknown primary cancer site, and 5 patients had other cancer sites, including renal cell and colorectal cancer.

The cpr documentation of the patients was analyzed according to patient characteristics. The number of metastasis sites was nonsignificant between patients with and without documented cpr code status (*p* = 0.22). No specific metastasis site was significant for documentation of cpr code status, including bone, brain, liver, lung, soft tissue, and lymph nodes.

As compared with patients who had no documentation of cpr code status, patients with a documented status were significantly older (mean age: 74.8 years vs. 67.4 years; *p* = 0.03). Patients with a documented status also had a significantly poorer kps score (median: 40 vs. 70; *p* = 0.0001). We observed no statistically significant difference in documentation of cpr-related advance directives for men and women (*p* = 0.82).

**Figure 2:**
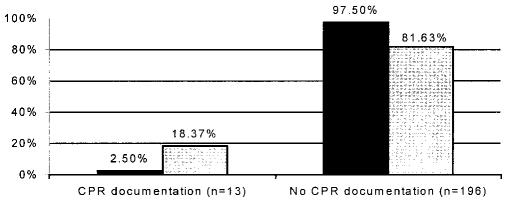
Frequency of cardiopulmonary resuscitation (cpr) code status documentation among inpatient (shaded bars) and outpatient (black bars) subpopulations.

We also tested differences in cpr documentation between the inpatient and outpatient subpopulations. Inpatients had significantly more documented references to cpr code status than did outpatients (*p* = 0.0004). Of the patients with documented references to cpr code status, 69.2% (9 of the 13 patients) were inpatients and 30.8% (4 of the 13 patients) were out-patients. Only 4 of the 160 outpatients (2.5%) had documentation of cpr code status upon arrival at the rrrp or bmc, but 9 of the 49 inpatients (18.37%) had reference to cpr status in their medical records at presentation to the clinics (Figure 2).

Differences between the inpatient and outpatient groups were also tested according to patient characteristics, including age, kps score, and primary cancer site. Inpatients (*n* = 49) had significantly poorer performance status as compared with outpatients (median kps score: 50 vs. 70; *p* < 0.0001). The differences in all other characteristics, including age and primary cancer site, were nonsignificant between the inpatients and the outpatients.

## 4. DISCUSSION

Life-sustaining interventions through cpr or chest compressions can be futile and non-beneficial to cancer patients with extensive disease and poor prognosis; these interventions fall outside the provision of best standard care. Fewer than 25% of patients who undergo cpr are resuscitated^2^.

The cpr code status is a difficult subject for discussion with terminally ill cancer patients and their family members. However, if such discussions occur or if any decisions are made regarding the withholding of potentially life-sustaining treatments at the end of life, proper documentation is essential to protect patient autonomy and to ensure that advance directives are followed according to the wishes of the patient and the family.

Patients and caregivers make end-of-life decisions in advance in the hopes that they will be at peace and experience closure and dignity during the last days, regardless of where the patient arrests—at home, in hospital, or in an ambulance. However, without clear and proper documentation, end-of-life decisions and the dignity of the patient may be compromised because of a simple, easily avoided miscommunication.

A survey about the perception of dnr orders and how and when to present the necessary information was administered to 23 cancer patients. The qualitative analysis of the survey found that most patients preferred written rather than verbal communication of dnr orders so as to avoid miscommunication and to highlight the importance of maintaining autonomous rights^12^. Policy at swchsc states that decisions about whether life support is right for a patient should be clearly noted on the patient’s medical health record so that all health care providers involved are aware of the decisions. Also, if patients are transferred from one facility to another or from one wing in the hospital to another, the policy specifies that the patient’s cpr status be reviewed and clearly identified.

If dnr orders are written in outpatient settings or at other institutions, all health care providers should be aware of the situation, whether they are providing care in the same institution or elsewhere. Communication, discussion, and clear documentation for all care providers attending to the patient are essential to ensure that the patient’s wishes are respected and that dignity is maintained during the dying process.

If cpr code status is identified in the medical records, oncologists providing outpatient palliative radiotherapy can easily confirm dnr orders with the patient or family, rather than assume full code against the wishes of the patient. Although few or no situations involving patient cardiac arrest have occurred in the rrrp or bmc clinics or during palliative radiation treatments, proper documentation of code status is still imperative, because a chance of arrest in these patients remains.

We identified no studies in the literature that report or audit documentation patterns of dnr decisions for outpatient palliative cancer clinics. A survey of 203 radiology programs in the United States concerning compliance with dnr orders for hospital in-patients transported to outpatient radiology departments demonstrates the difficulties that ambiguous documentation of dnr orders can cause in the outpatient setting^13^. Hospital-based radiology departments were used to represent and test concerns about insufficient documentation and knowledge of cpr practices by staff in regard to the dnr status of patients transported to hospital locations outside their inpatient room and ward. Of the 248 radiology personnel responding, 24% initiated cpr for patients with dnr orders, and almost 40% admitted to having resuscitated patients with dnr orders in the past. Like the personnel in a radiology department, personnel in our outpatient radiotherapy clinics are temporarily responsible for a patient’s care, and if they have unclear or limited information about the patient’s cpr status, they may not be able to appropriately respond to a life-threatening decline.

In our small cohort with documented advance directives, details of the documentation were ambiguous. It was unclear who made the advance directive decision, which is consistent with studies in the literature. Specifically, a study of 333 inpatients with written dnr orders in a 450-bed community hospital in the United States found that, in 45% of cases, the orders contained no documentation of who participated in the dnr decision^5^.

In our study, the dnr order was often difficult to locate in the records of our patient cohort; it might be written in the middle of a long physician progress note or handwritten in the back of the inpatient hospital chart. Also, although 8 patients with documented cpr code status arrived at the clinics by ambulance, only 4 had documentation of their cpr code status on the ambulance transfer form.

Guru, Verbeek, and Morrison conducted a retrospective review^14^ of out-of-hospital cardiac arrest patients who were described as having a terminal illness by paramedics that serve the Department of Emergency Services at swchsc; those authors compared resuscitation efforts in patients with and without a dnr request. Patients were included if clearly documented descriptive terms were used for the palliative and incurable nature of the disease and if a clear definition of a dnr request was present. During the 10-month study period in 1996–1997, 144 of 1534 calls for non-accidental cardiac arrest involved patients with a terminal illness. Descriptions of the type of illness were not provided. The mean age of patients was 72.2 years (range: 11–101 years). A dnr request was present in 62.5% of cases. Caregivers verbally made the dnr request in 70% of cases; only 30% of cases had actual dnr requests in written format. Ethical difficulties arise in such situations: Even when the family verbally requests the withholding of a life-sustaining intervention from a patient with an underlying incurable illnesses who has chosen to die at home and undergo cardiac arrest, emergency paramedics are forced by policy to attempt resuscitation if a *written* order is not available^15^.

Mandatory resuscitative efforts, in situations of non-beneficence and against the wishes of the patient and family, can be avoided with clear documentation of advance directives. Paramedics and caregivers strongly agree that the creation of an out-of-hospital dnr protocol should be established to maintain dignity at the end of life^14^. Many studies have been conducted in the United States on the creation of a prehospital dnr request for outpatients^16^–^27^. However, despite the interest and need identified by patients, families, and health care professionals, there are currently no published Canadian policies, and no jurisdiction has, to our knowledge, developed an dnr protocol outpatient^14^.

In our study, patients with documented cpr code status were significantly older and had a poorer performance status score than those lacking documentation. That finding is expected and consistent with the literature, which indicates that advanced age is an important independent determinant of survival following resuscitation, as are multiple comorbidities or concomitant disease and patient dependency on others^28^,^29^.

The differences in the documentation of cpr code status among inpatients as compared with that among outpatients can be attributed to differences in patient characteristics. As expected, inpatients of hospitals, hospices, or nursing homes had poorer performance status scores than did outpatients who had maintained the mobility and independence to live at home. Inpatients of health care institutions are also more likely to be approached with the topic of dnr orders and cpr code status than are patients at home who are attending outpatient palliative clinics.

Although the median life expectancy of patients seen in the rrrp is approximately 4 months, meta-static cancer patients are a heterogeneous group ranging from those who are ambulatory and independent, to those who are moribund and confined to bed. Most patients seen at our outpatient palliative radiotherapy clinics maintain their ability to attend the clinic, have better performance status than inpatients, and tend to have a better prognosis than inpatients from a hospice. Breast or prostate cancer patients with bone-only metastases can live for many years with their illness, and they therefore may not have had discussions about or have arranged for documentation of cpr code status.

The present study is limited by the small sample size in the group of patients with documented cpr advance directives (*n* = 13). However, despite the small sample, our findings clearly demonstrate that only a very small proportion of symptomatic, advanced cancer patients referred for palliative radiotherapy have any documentation of cpr code status. Proper documentation is essential in the event of cardiopulmonary arrest and is especially important when new patients are initially referred for palliative radiation to facilities where members of the health care team may not be familiar with the patient’s medical history or wishes regarding cpr.

Because of the small sample, details regarding the cpr decisions were also ambiguous. Furthermore, it was not possible to ascertain the proportion of patients without advance directives who were ultimately subjected to futile cpr.

More-detailed information on physician characteristics could be collected in future studies. A survey of the documentation practices in a 450-bed community hospital in San Francisco concluded that documentation of dnr decisions may be related more to physician characteristics than to the terminal status of the patient^5^. Lipton concluded that detail in dnr documentation is related both to physician medical specialty (oncologists being more likely to write dnr orders) and to the number of years in practice (physicians in practice less than ten years being more likely to write more detailed notes on dnr orders).

Questions remain about why patients have no documented cpr code status in their medical records, especially when attending an outpatient palliative clinic for symptom control. Was the dnr issue ever raised with the patient? Many physicians do not receive adequate training in discussions surrounding the end of life and may be reluctant and uncomfortable to hold such discussions with terminally ill patients and their families. Was dnr discussed at a time when the patient was not ready to decide, or when the patient did not have the cognitive and mental capacity to make such a profound decision? In the context of a multidisciplinary palliative cancer care, it is important to consider who should be responsible to initiate discussion of cpr advance directives and end-of-life treatment decisions, and what is the best time, context, and situation to start the discussion. It may also be beneficial to consider focusing on patients with no documented code status to determine contributing variables.

Based on the results of the present study, we are considering modifications to our standardized referral forms to the rrrp so as to improve communication between referring physicians and radiation oncologists. It would be useful to have standardized cpr directive forms that would apply across various health care institutions and outpatient settings and during ambulance transfers. We also recommend the use of a standardized order form across Ontario—one that would apply across various health care institutions and out-of-hospital settings.

Standardized order forms have been shown to be successful in the United Kingdom to complement progress note documentation and to improve clarity in recording this significant decision. Butler *et al.* conducted a retrospective audit of 94 case notes with dnr decisions 30,31. After these authors noted poor documentation details in the study sample, a standardized order form was implemented to complement case notes. Subsequently, a prospective re-audit was performed. In the initial audit, 86.2% of dnr decisions were found to be in keeping with accepted guidelines as outlined by the U.K. policy on resuscitation. In the re-audit after implementation of the standardized order form, a significant improvement to 98.4% was reported (*p* < 0.01)^30^.

A survey of physicians who requested and obtained a supply of North Carolina’s out-of-facility dnr forms in 1993 found that, for dnr orders, 98% of respondents supported a single, universal form that would apply across all health-care settings, including outpatient settings, ambulance transfers, and hospitals^27^. Another study in California found that a procedure-specific dnr order form can improve documentation of dnr decisions^32^.

Many studies recommend the use of a standardized dnr order form with detailed documentation indicating who made the decision, which physician documented the decision, and the exact interventions that the patient does and does not want. A detailed form of this kind provides accurate and complete documentation, eases treatment decisions for patients, and makes the topic of dnr more approachable for physicians who need to initiate this discussion with patients^33^–^35^.

Further development is required to improve communication of dnr orders among health care providers caring for the terminally ill. We suggest considering the implementation and use of a centralized electronic patient database system that, if accessible to “treating” physicians, may also encourage more consistent documentation and better communication regarding dnr orders.

## 5. CONCLUSION

Few patients referred for outpatient palliative radiotherapy had any documentation of cpr code status in their accompanying medical records. Patients with a documented code status were significantly older, had a poorer performance status score, and were more likely to be inpatients of a hospital, hospice, or nursing home. Proper documentation of cpr-related advance directives is essential in the event of cardio-pulmonary arrest and is especially critical in an out-patient palliative radiotherapy clinic, where a new patient may not be well known to the health care providers. In the future, we may implement identification of patient cpr code status on the referral form for the rrrp and the bmc. We recommend the use of standardized order forms applicable both to in-hospital and out-of-hospital settings.

## Figures and Tables

**Table I:** Patient characteristics (*n* = 209)

Sex [*n* (%)]	
Male	109 (52)
Female	100 (48)
Age at consultation (years)	
Median	70
Range	28–96
Patient origin [*n* (%)]	
Home (outpatient)	160 (76.6)
Hospital, hospice, or nursing home (inpatient)	49 (23.5)
Arrived by ambulance [*n* (%)]	51 (24.4)
Primary cancer sites [*n* (%)]	
Lung	67 (32.1)
Breast	48 (23.0)
Prostate	45 (21.5)
Colorectal	15 (7.2)
Unknown	11 (5.3)
Renal	10 (4.8)
Others	13 (6.2)
Site of metastasis [*n* (%)]	
Multiple sites	121 (57.9)
Bone	160 (76.6)
Lymph nodes	56 (26.8)
Brain	49 (23.4)
Lung	44 (21.1)
Liver	39 (18.7)
Soft tissue	37 (17.7)
Adrenal glands	11 (5.3)
Karnofsky performance status score at consultation	
Median	70
Range	20–100

**Table II:** Details of cardiopulmonary resuscitation (cpr) code status documentation (*n* = 13)

*Documentation variable*	*[*n*(%)]*
cpr code status	
Do not resuscitate (dnr) coded	8 (61.5)
Full code (cpr)	5 (38.5)
Location of documentation in the medical records^a^	
Physician progress notes	7 (53.8)
Inpatient hospital chart	5 (38.5)
Ambulance transfer forms	4 (30.8)
Physician who provided the documented reference	
Medical oncologist	5 (38.5)
Palliative care physician	1 (7.7)
Family physician or general practitioner	1 (7.7)
Hospital internist in the emergency room	1 (7.7)
Unknown physician category	5 (38.5)
Person making the decision	
Patient	3 (23.1)
Surrogate decision maker (family member)	2 (15.4)
Unknown	8 (61.5)

a cpr code status may have been documented in multiple medical records for a given patient. A total of 16 locations were identified for the 13 patients.
